# Medium-term Electrophysiologic Effects of a Cellularized Scaffold Implanted in Rats After Myocardial Infarction

**DOI:** 10.7759/cureus.2959

**Published:** 2018-07-10

**Authors:** Theofilos M Kolettis, Eleni Bagli, Eleonora Barka, Dimitrios Kouroupis, Marianthi Kontonika, Agapi D Vilaeti, Maria Markou, Maria Roumpi, Violetta Maltabe, Vassilios La Rocca, Simeon Agathopoulos, Theodore Fotsis

**Affiliations:** 1 Cardiology, Cardiovascular Research Institute, Ioannina, GRC; 2 Division of Biomedical Research, Institute of Molecular Biology and Biotechnology – Forth, Ioannina, GRC; 3 Ceramics and Composites Laboratory, Materials Science and Engineering, University of Ioannina, Ioannina, GRC; 4 Cardiology, Cardiovascular Research Institute, Athens, GRC; 5 Division of Biomedical Research, Institute of Molecular Biology and Biotechnology – Forth, Ioannina, GRC; 6 Ceramics and Composites Laboratory, Science and Engineering, University of Ioannina, Ioannina, GRC

**Keywords:** myocardial infarction, cardiac repair, mesenchymal stem-cells, alginate-scaffold, ventricular arrhythmias, conduction-delay, repolarization-dispersion

## Abstract

Background

Cardiac repair strategies are being evaluated for myocardial infarctions, but the safety issues regarding their arrhythmogenic potential remain unresolved. By utilizing the in-vivo rat model, we have examined the medium-term electrophysiologic effects of a biomaterial scaffold that has been cellularized with spheroids of human adipose tissue, derived from mesenchymal stem cells and umbilical vein endothelial cells.

Methods

Mesenchymal stem cells, which exhibit adequate differentiation capacity, were co-cultured with umbilical vein endothelial cells and were seeded on an alginate based scaffold. After in-vitro characterization, the cellularized scaffold was implanted in (n=15) adult Wistar rats 15 min post ligation of the left coronary artery, with an equal number of animals serving as controls. Two weeks thereafter, monophasic action potentials were recorded and activation-mapping was performed with a multi-electrode array. An arrhythmia score for inducible ventricular tachyarrhythmias was calculated after programmed electrical stimulation.

Results

The arrhythmia score was comparable between the treated animals and controls. No differences were detected in the local conduction at the infarct border and in the voltage rise in monophasic action potential recordings. Treatment did not affect the duration of local repolarization, but tended to enhance its dispersion.

Conclusions

The fabricated bi-culture cellularized scaffold displayed favorable properties after in-vitro characterization. Medium-term electrophysiologic assessment after implantation in the infarcted rat myocardium revealed low arrhythmogenic potential, but the long-term effects on repolarization dispersion will require further investigation.

## Introduction

Myocardial infarction (MI) is the leading cause of chronic heart failure worldwide. Because of its vast burden on society, novel interventions that aim to ameliorate post-MI left ventricular (LV) dilatation and dysfunction are being intensely investigated [1]. The initial regenerative efforts focussed on transplanting skeletal myoblasts directly into the myocardium, an approach that is quickly surfacing in small-scale clinical trials [2]. However, concerns were raised regarding the arrhythmogenic potential of such therapy, especially in patients already at risk of ventricular tachyarrhythmias (VTs) [3]. As a result, skeletal myoblasts have largely been substituted by other cell types, such as bone marrow or non-bone-marrow derived mesenchymal stem cells (MSCs); the latter are particularly attractive as they can be easily harvested and are readily available for transplantation into patients, shortly after hospitalization for acute MI. In view of previous reports, newer studies have addressed the safety issues associated with MSC transplantation, but hitherto data have been inconsistent, indicating increased [4-5], neutral [6-7], or perhaps decreased [8-9] arrhythmogenic potential.

Over the past decade, the integration of cellular transplantation with biomaterials has broadened the horizon of therapeutic strategies for the prevention of LV remodeling and subsequent heart failure [10]. Biodegradable hydrogels may act as an extracellular matrix, thereby decreasing LV wall stress [11-12]; furthermore, the encapsulation of various cell types into polysaccharide structures enhanced cell retention post transplantation in animal models [13]. Based on these characteristics, coupled with the feasibility of catheter delivery, such therapies are progressing rapidly, with several clinical trials currently underway. However, previous studies have also cautioned against possible conduction abnormalities after biomaterial implantation that could alter the electrophysiologic milieu and predispose to VTs [14-15]. Moreover, safety concerns regarding cellular- or biomaterial-based therapies may be particularly relevant in case of their combined use, but this issue remains unresolved, as electrophysiologic end-points have been examined after implantation of either cells [4-8,16] or biomaterials [14-15,17].

In the present work, we have fabricated a biomaterial scaffold, characterized by a dynamic viscosity that is suitable for smooth delivery, coupled with optimal gelation properties; it was seeded with human adipose tissue (hAT)-derived MSCs and umbilical vein endothelial cells (HUVECs), thereby enhancing its angiogenic properties. The main aim of the present work was to evaluate the medium-term electrophysiologic effects of this cardiac repair approach in the rat MI model. For this purpose, we have examined the inducibility of VTs by programmed electrical stimulation (PES) two weeks post implantation; in addition, we recorded monophasic action potentials (MAPs), and we investigated local conduction and repolarization, by means of activation mapping.

## Materials and methods

Human adipose tissue mesenchymal stem cells

After informed consent was collected, human subcutaneous adipose tissue was harvested from adult donors undergoing elective surgical procedures; the human adipose-derived mesenchymal stem cells (hAT-MSCs) were isolated using collagenase I (Worthington, Lakewood, NJ, USA) and diluted in Dulbecco’s phosphate buffered saline (PBS) and bovine serum albumin (Sigma Aldrich, St. Louis, MO, USA) for four hours. The cell digests were seeded in 100 mm round plates in adipose-derived stem cells (ADSC) growth medium (Lonza, Cambridge, UK) at a cell density of 1 x 106. When 80% of confluent adherent cells were trypsinized, they were passaged at a one-to-one ratio, and the number of viable cells was evaluated using trypan blue (Invitrogen, Waltham, MA, USA).

Phenotypic characterization and tri-potentiality of hAT-MSCs

The cultured hAT-MSCs were characterized at the third passage by evaluating the expression of markers specific for mesenchymal stem cells (MSCs) and endothelial lineages (Table 1). During the flow cytometry (CyFlow, Partec, Münster, Germany), viable cells (stained with PE-conjugated antibodies) were identified, aided by 2 μg/ml of 7-amino actinomycin D (Invitrogen, Thermo Fisher Scientific, Waltham, MA, USA). The cultured MSCs were expanded and then subjected to differentiation induction protocols: during osteogenic differentiation, a semi-quantitative assessment was achieved by alizarin red S staining (Sigma Aldrich, Merck KGaA, Darmstadt, Germany). Assessment of the chondrogenic differentiation was carried out using toluidine blue (Sigma Aldrich, Merck KGaA, Darmstadt, Germany), and adipogenic differentiation using oil red stain (Sigma Aldrich, Merck KGaA, Darmstadt, Germany).

**Table 1:** Conjugated antibodies

Antibody name	Clone	Working dilution	Manufacturer
FITC donkey α-mouse	Polyclonal	1:200	Jackson ImmunoResearch Laboratories, Pennsylvania, USA
CD29-PE	MEM-101A	5μl neat	Immunotools, Friesoythe, Germany
CD31-PE	MEM-05	5μl neat	Immunotools, Friesoythe, Germany
CD44-PE	MEM-85	5μl neat	Immunotools, Friesoythe, Germany
CD90-PE	F15-42-1	5μl neat	AbD Serotec, Puchheim, Germany
CD105-PE	MEM-226	5μl neat	Immunotools, Friesoythe, Germany
CD146-PE	P1H12	5μl neat	BD Biosciences, Oxford, UK
IgG1-PE	PPV-06	2.5μl neat	Immunotools, Friesoythe, Germany
IgG2-PE	PPV-04	2.5μl neat	Immunotools, Friesoythe, Germany

Co-culture spheroids

The hAT-MSCs and HUVECs were labeled with green (PKH67, Sigma Aldrich, Merck KGaA, Darmstadt, Germany) and red (PKH26, Sigma Aldrich, Merck KGaA, Darmstadt, Germany) fluorescent, respectively, and were suspended in an endothelial cell growth medium (EGM2)  (Lonza, Basel, Switzerland), containing carboxymethylcellulose substrate. Spheroids were formed by the hanging drop culture technique, as follows: 10 μl of cell suspension, containing 900 HUVECs and 100 hAT-MSCs, were hanged from the lid of a 100 mm petri dish, forming a single spheroid per drop, imaged under confocal microscopy (TCS-SP5, Leica Microsystems, Wetzlar, Germany).

In vitro angiogenesis assay and immunofluorescence

The HUVEC/hAT-MSC spheroids were seeded on each well of matrigel-coated slides and were cultured for five days. Sprouts grown from each spheroid were observed daily under phase contrast (Axiovert 100, Carl Zeiss Microscopy, Thornwood, NY, USA) and confocal microscopy. The third passage hAT-MSCs were fixed with paraformaldehyde, followed by permeabilization with Triton X-100 (Sigma Aldrich, Merck KGaA, Darmstadt, Germany) for four minutes. After blocking with fetal calf serum, the cells were stained with primary antibodies for one hour (Table 2); after the addition of secondary antibodies, imaging was performed with confocal microscopy.

**Table 2:** Purified antibodies

Antibody name	Clone	Working dilution	Manufacturer
FITC donkey α-goat	Polyclonal	1:200	Jackson ImmunoResearch Laboratories, Pennsylvania, USA
αSMA	1AL	1:200	Biogenex, California, USA
Calponin	Mouse monoclonal	1:100	M3556 Dako, Agilent, CA, USA
SM22α	Rabbit polyclonal	1:500	ABCAM, Cambridge, United Kingdom
NG2	Rabbit polyclonal	1:100	AB5320, Millipore, Burlington, Massachusets, USA
FITC-CD44		1:100	Immunotools, Germany
FITC-CD105		1:100	Immunotools, Germany

Fabrication of the alginate scaffold and encapsulation

The scaffold was a mixture of high and low molecular weight alginate. For the former, sodium alginate powder with a high content of guluronic units (FMC Biopolymers, Oslo, Norway) was used, whereas the low molecular weight alginate was prepared by acidic hydrolysis. After oxidation with sodium periodate (Sigma Aldrich, Merck KGaA, Darmstadt, Germany) and purification, the solidified product was characterized using Fourier-transform infrared (FTIR)-spectroscopy (Jasco, Easton, MD, USA). Subsequently, covalent grafting with an oligopeptide (Eurogentec, Seraing, Belgium) was carried out, following aqueous carbodiimide chemistry. A mixture (50:50 ratio) of arginine-glycine-aspartic acid (RGD) oxidized high and low molecular weight alginate was dissolved in sodium chloride (NaCl) solution; after freeze-drying, a highly porous scaffold was obtained, with an average pore diameter of ~100 μm, measured by mercury porosimetry (PM33GT, Quantachrome, Kawasaki City, Japan). The aqueous solution of the alginate mixture was stored in 0.12 ml aliquots, containing 100 methylcellulose drops, each of 1000 cells (at HUVΕCs: MSCs of 9:1).

Viscosity

The viscosity of the liquid scaffold was measured (Bohlin Visco-88, Malvern Instruments, UK), followed by calculations of density functional theory at various calcium ion (Ca2+) concentrations. Such measurements provided an accurate assessment of the maximum dynamic viscosity after phase transition into a hydrogel, in experimental conditions resembling those after intra-myocardial injection.

Animal cohort and experimental protocol

The in-vivo experiments were conducted on 36 Wistar rats (17-20 weeks of age, weighing 283 ± 5g). The animals were housed in plexiglass cages, with free access to water and a standard pellet diet. Housing conditions were kept optimal, in terms of temperature, humidity, and light-to-dark cycles. The investigation conforms to national (56/106/2013) and European (2010/63/EU) legislation. Low dose (2.5mg/kg/day) cyclosporine A was administered by gavage to all animals, commenced on the day prior to MI induction and continued throughout the study; this regimen provides effective immunosuppression, without affecting the myocardium. MI was generated, and the scaffold was implanted 15 minutes thereafter. Beginning on the day following ligation, enalapril (30mg/kg per day) was given throughout the study, as previously [18].

Three groups were randomly formed, namely six sham-operated rats, in which no MI was induced and no treatment was administered; controls (n=15), in which MI was induced, followed by normal saline intra-myocardial injections; and treated rats, in which MI was induced, followed by cellularized scaffold implantation (n=15). Mortality was recorded during twice-daily inspections for two weeks, followed by an electrophysiologic assessment. All procedures and subsequent analyses were performed in a blinded fashion.

Induction of myocardial infarction and scaffold implantation

The rats were intubated and mechanically ventilated (respirator model: 7025, Ugo Basile, Varese, Italy) at 85 breaths/min; anesthesia was maintained with a mixture of oxygen and 2.5% sevoflurane (Abbott Laboratories, IL, USA). The left coronary artery was ligated midway between its origin and the apex in a consistent manner, ensuring a comparable ischaemic area in all animals; the sham operation consisted of encircling but not ligating the artery. MI was confirmed by ST-segment elevation in a six-lead electrocardiogram (ECG) (QRS Card, Pulse Biomedical, Norristown, PA, USA) after amplification by software (Cardiology Suite, Pulse Biomedical, PA, USA). To further validate the comparability between MI groups, infarct size was measured in three randomly chosen animals from each MI group, 24 hours post ligation. For this purpose, the animals were euthanized with potassium chloride under sevoflurane anesthesia; the heart was excised, frozen, cut into 2 mm slices, incubated (in triphenyltetrazolium chloride for 15 minutes) and fixed (in 10% formalin for 20 minutes). The slices were scanned and the areas of infarcted and non-infarcted myocardium were measured, using the image tool software (University of Texas, USA), and the infarct size was derived as the ratio between the infarcted and total LV volume.

The cellularized scaffolds were implanted by intra-myocardial injections around the ischemic border, as before [11-12]. The incision was then closed in three layers and pneumothorax was evacuated; for analgesia, a single intraperitoneal injection of an opioid analgesic (buprenorphine, 0.05mg/kg) was administered postoperatively. Two weeks post MI, the rats were anesthetized, as described above, for electrophysiologic assessment.

Microwave anisotropy probe (MAP) recordings

Recordings were performed with a hand-held MAP probe (EP Technologies, Boston Scientific Corporation, Marlborough, MA, USA) connected to a preamplifier (EPT). Data were streamed into a personal computer, equipped with an analog-to-digital converter (BNC-2110, National Instruments, NI, Austin, Texas, USA) and a data acquisition card (DAQ-6023E, National Instruments, Austin, TX). The signals were filtered and were recorded with a software program, custom written in LabView (National Instruments, Austin, TX). The MAP probe was placed on the LV base and was gently shifted toward the infarct area, until an abrupt transition to low amplitude signals was observed; it was then withdrawn slowly, until the re-emergence of a good quality MAP signal, thereby ensuring recordings from the border zone surrounding the infarct scar. Recordings from the lateral right ventricular (RV) myocardium were also obtained for reference. Data analysis included action potential duration (APD) at 90%, 75%, 50%, and 25% of repolarization. In addition, depolarization was examined by the maximum value of voltage rise (dV/dtmax), an important determinant of conduction velocity, reflecting the availability of sodium channels at the onset of depolarization.

Multi-electrode array mapping

We used a 32-electrode array, selected from a commercially available device (FlexMEA72, Multi-Channel Systems, Reutlingen, Germany); it consists of titanium nitrate electrodes and golden contact pads mounted on polyimide foil, yielding a 50 kΩ impedance. The electrodes were arranged in a 4x8 configuration, with 1.25X1.50 mm inter-electrode distance. The array was connected via an adaptor (ADPT-FM-72, Multi-Channel Systems, Reutlingen, Germany) to two noise rejecting, shielded connector blocks (SCB-68A, 782536-01, National Instruments, Austin, TX). Data were continuously streamed into an acquisition system (PCI-6289, M-Series-DAQ, National Instruments, Austin, TX) via two shielded cables (SHC68-68-EPM, National Instruments, Austin, TX). Ventricular electrograms were recorded in reference to Wilson’s central terminal; the selected unipolar configuration permits easier identification of repolarization, a feature particularly useful in rodent models. As before [19], the multi-electrode array was placed on the anterior LV epicardium in a consistent manner, guided by anatomical landmarks, and reference values were obtained from respective RV sites. The recording was made at a sampling frequency of 5 kHz, using another software program custom-written in LabView; the analysis options of the software provide measurements aided by automated point recognition. To enhance the accuracy of measurements, values at each recording channel were calculated as the average of 10 successive sinus electrograms.

Ventricular conduction was evaluated by the activation delay, i.e., the time difference between the initial dV/dtmin of the first and last electrograms obtained in each set of 32 channels. The activation recovery interval (ARI) was measured as the interval between the electrogram onset and the dV/dtmax of the T wave; such a definition was favored over the end of the T wave, as this part likely reflects remote repolarization. To account for the effects of heart rate (HR) on repolarization, corrected values were calculated, using the formula ARIc=ARI/(RR/150)1/2. Lastly, repolarization dispersion was assessed with the use of the intra-ventricular repolarization heterogeneity index (μ), as ARI95-ARI5/ARI50, using the 95th and fifth percentiles and median values of the distribution in the array, respectively. Repolarization dispersion was illustrated by the construction of isochronal maps.

Programmed electrical stimulation

The RV outflow tract was paced via a custom-made, bipolar hook electrode, kindly provided by Professor Y. Etzion (Ben-Gurion University of the Negev, Israel); the design of the electrode permits accurate positioning on the epicardium without suturing, offering an excellent pacing threshold. A three-lead ECG (PBI) was continuously recorded throughout the procedure. VT induction was attempted with up to three extrastimuli after a basic drive of 20 paced beats at a cycle length of 100 msec; no distinction was attempted between ventricular tachycardia and fibrillation, collectively referred to as VTs. The severity of induced VTs was quantified by an arrhythmia score, as shown in Table 3.

**Table 3:** Arrhythmia score

score	outcome
0	noninducible
1	nonsustained ventricular tachycardia (6-14 beats) induced with triple extra stimuli
2	sustained ventricular tachycardia (>15 beats) with triple extra stimuli
3	nonsustained ventricular tachycardia with double extra stimuli
4	sustained ventricular tachycardia with double extra stimuli
5	nonsustained ventricular tachycardia with single extra stimulus
6	sustained ventricular tachycardia with single extra stimulus
7	ventricular tachycardia induced during the basic drive
8	fatal ventricular tachycardia during the basic drive

Statistical analysis

Values are presented as mean ± standard error of the mean (SEM). Kaplan Meier survival plots were constructed for each group, and the two MI groups were compared with the Peto & Peto Wilcoxon test. Comparisons between the two categorical variables were made with Fisher exact two-tailed test and between two continuous variables with student’s t-test. Pearson’s product-moment correlation coefficient (r) is reported in correlations between two continuous variables. Differences in continuous variables between the three groups were assessed with one-way analysis of variance, followed by the post-hoc Duncan’s multi-stage test. Statistical significance was defined at p<0.05.

## Results

Cellular cultures and spheroid construction

**Figure 1:**
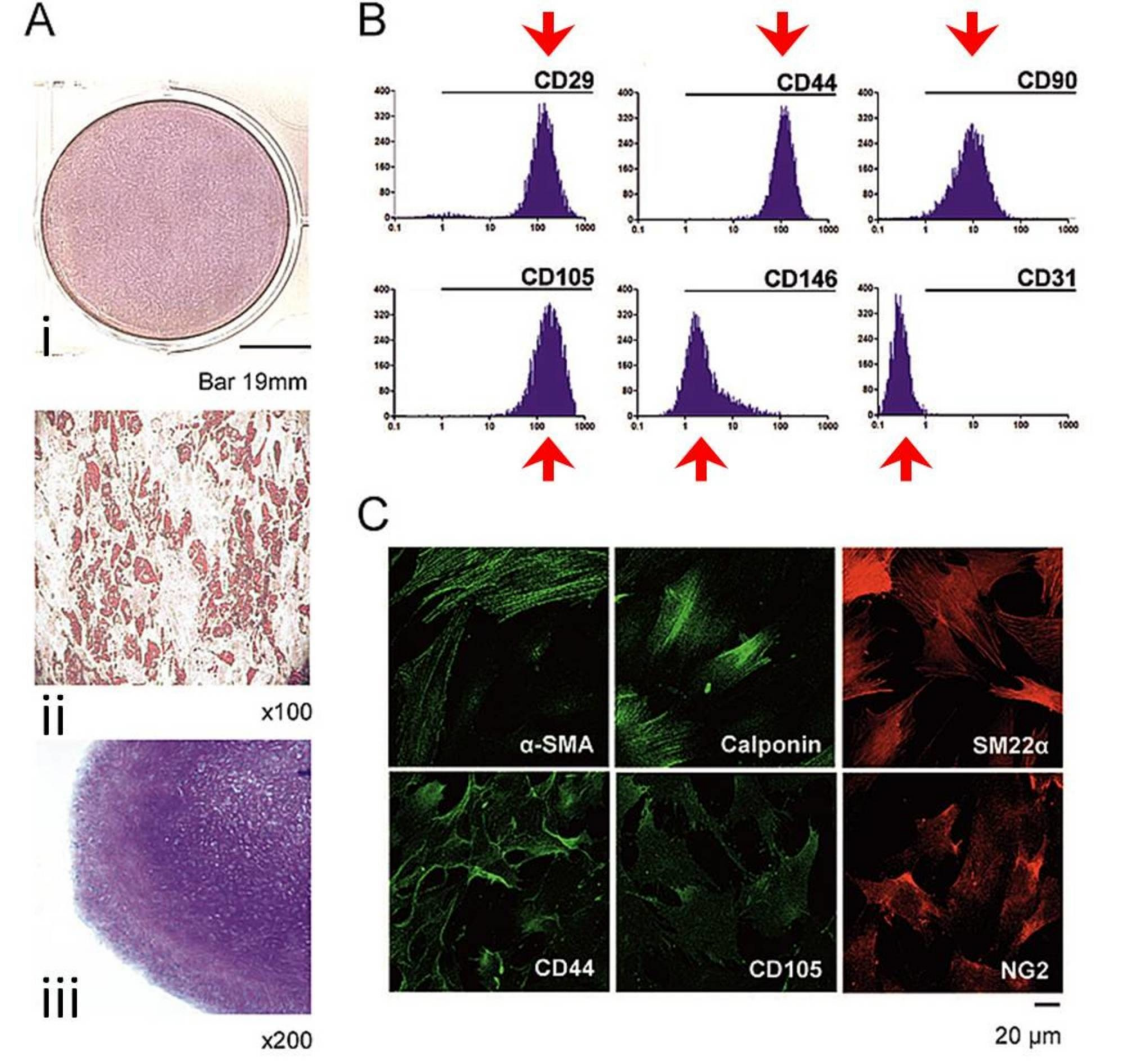
Cultured mesenchymal stem cells (A): Mesenchymal stem cells displayed differentiation capacity to (i) osteoblasts, (ii) adipocytes,  and (iii) chondroblasts; (B): The expression was low for CD31, but high for CD29, CD44, CD90, CD105, CD146. C) Likewise, high expression was present for myofibroblastic (α-smooth muscle actin [α-SMA], calponin, SM22α), mesenchymal (CD44, CD105), and pericytic (NG2) related markers.

Enzymatic processing of adipose tissue yielded primary hAT-MSC cultures that displayed tri-potential differentiation capacity. Specifically, in osteogenic conditions, the hAT-MSCs displayed high alkaline phosphatase activity (Figure 1Ai) whereas adipogenic induction resulted in lipid droplet cytoplasmic accumulation (Figure 1Aii) and chondropellet generation in chondrogenic conditions (Figure 1Aiii). The third passage cultures exhibited high positivity for MSC-related markers, but an absence of expression for CD31, the main endothelial marker (Figure 1B). Immunofluorescence analysis validated the expression of MSC, myofibroblastic, and pericytic related markers (Figure 1C).

**Figure 2:**
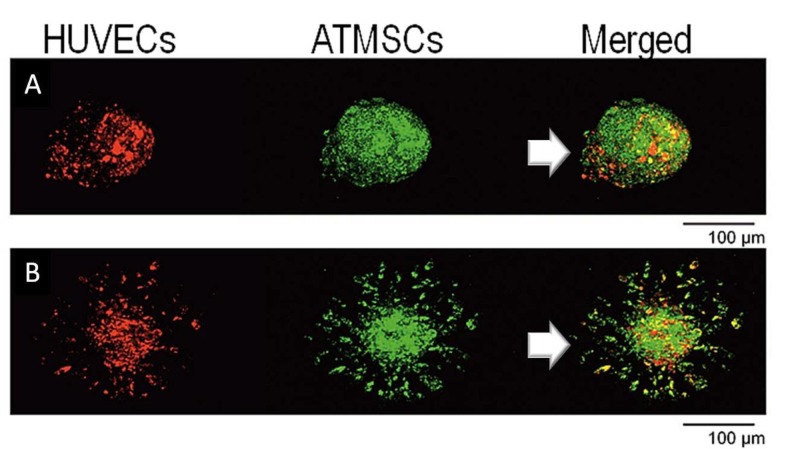
Spheroid construction Confocal microscopy images of mesenchymal and endothelial stem cells, located at the periphery of spheroids (A), displaying angiogenic potential (B)

By the fifth day, the HUVECs were located at the periphery of spheroids (Figure 2A), which displayed an average diameter of ~110μm. On a Matrigel matrix, hAT-MSC migration and tubular structure formation were observed; interestingly, the HUVECs followed a similar migration pattern by the second day, with co-localization with hAT-MSCs in most vascular sprouts (Figure 2B). This network expanded during the succeeding five days and remained stable for at least seven days thereafter.  

Scaffold viscosity

The viscosity of the alginate scaffold was 0.05 Pa×s in liquid form, at 37^o ^C. A maximum dynamic viscosity of 0.38 ± 0.02 Pa×s was calculated (at 1.78 Hz rotation frequency), reflecting that anticipated during the gelation phase in experimental conditions, mimicking intra-myocardial implantation.

Implantation, infarct size, and mortality

Implantation of the cellularized scaffold was successful in all rats. Infarct size was almost identical in the treated group (31.0 ± 1.8%) and controls (30.7 ± 2.1%). At the end of the two-week observation period, there were 20 survivors from the three groups, namely six sham-operated, six controls, and eight treated animals; mortality rates were comparable in the MI groups.

Heart rate (HR)

Two weeks post MI, HR was lower (p=0.02) in sham-operated animals than in controls, whereas no difference was present between treated rats and the remaining groups.

Induced arrhythmias

**Figure 3:**
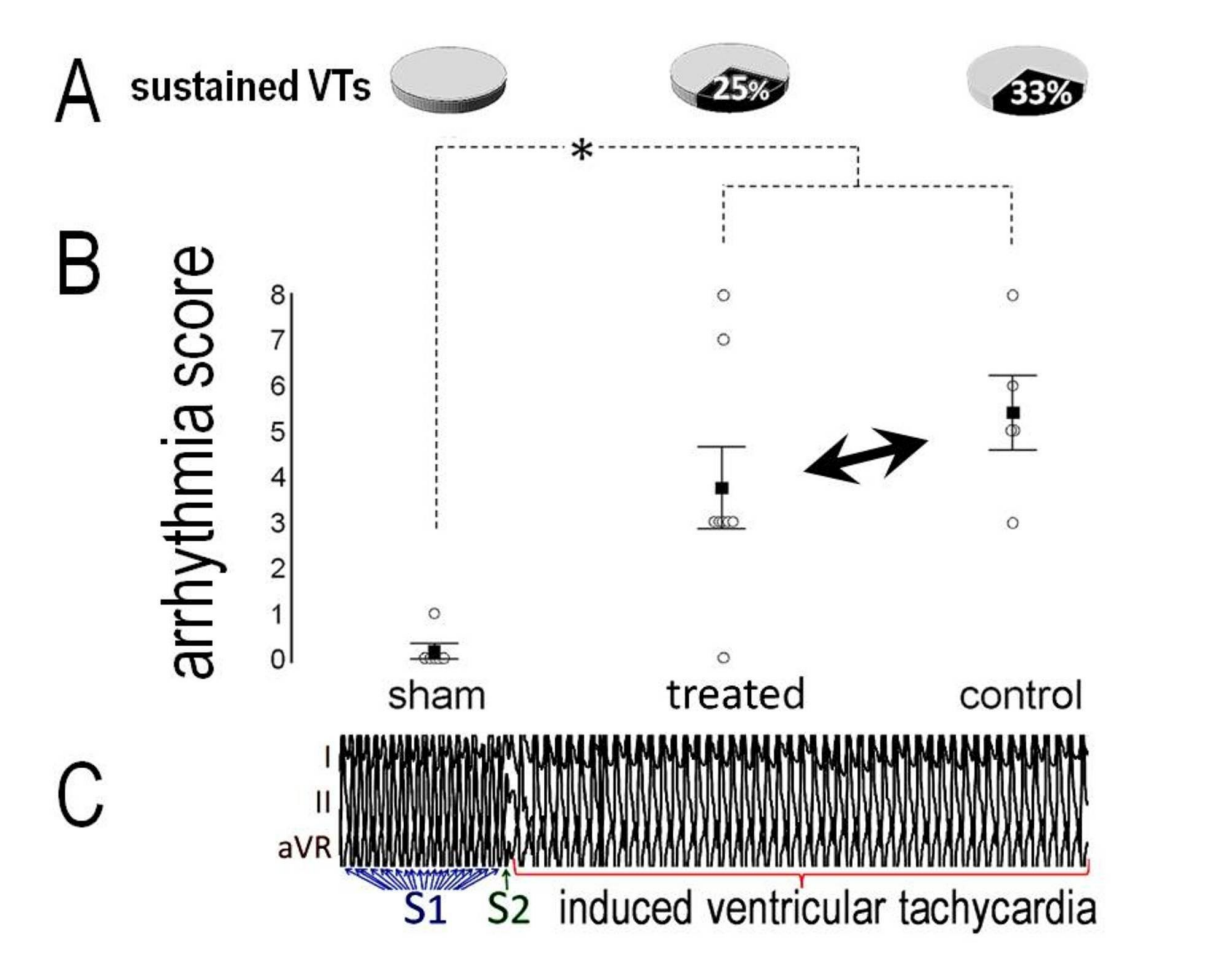
Induced ventricular tachyarrhythmias The incidence of ventricular tachyarrhythmias after programmed electrical stimulation (A) and the arrhythmia score (B) were comparable in treated rats and controls, and higher than after sham-operation (asterisk). (C): Example of induced ventricular tachycardia with one extra stimulus (S2) after 20 paced beats (S1).

No VTs were induced in the sham-operated group, except for a short burst in one rat. By contrast, sustained VTs occurred in two (25%) treated animals and in two (33%) controls (Figure 3A), with comparable incidence and overall arrhythmia scores between groups (Figure 3B). An example of induced VT is shown in Figure 3C.

Voltage rise and conduction at the infarct border

**Figure 4:**
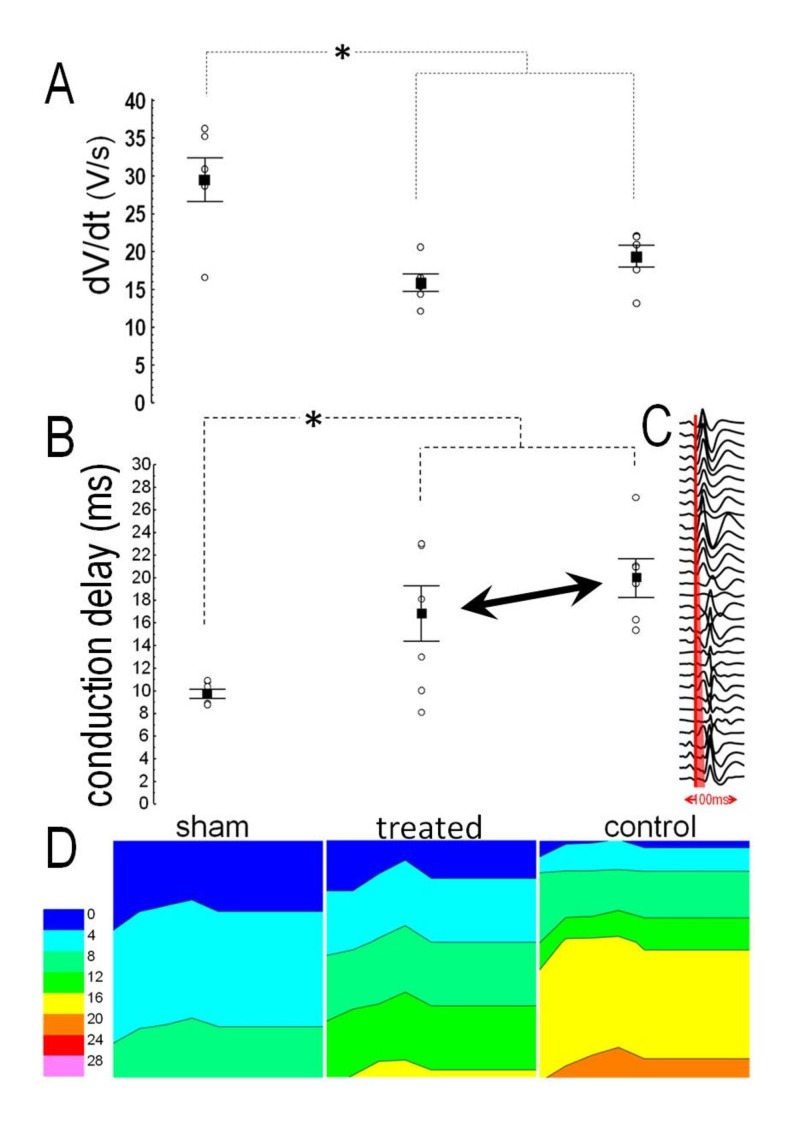
Local conduction (A): Voltage rise from monophasic action potentials was comparable in treated rats and controls, and lower than after sham-operation. (B): Conduction delay was comparable in treated rats and controls, and longer than after sham operation. (C): Examples of isochronal propagation maps. Asterisk denotes significant difference

Voltage rise (dV/dtmax) in MAP recordings from the infarct border was lower in either MI group than in sham-operated animals, with similar values between treated rats and controls (Figure 4A); no variance was present in the RV dV/dtmax. MI prolonged the conduction delay at the border zone, evidenced by lower values in sham-operated animals when compared to controls (p=0.002) or treated animals (p=0.015); however, no difference was present between the latter groups (Figure 4B). An example of local activation can be seen in Figure 4C, and representative examples of LV isochronal propagation maps from the three groups are shown in Figure 4D. In the RV, no variance was present in the conduction delay.

Repolarization duration and dispersion

**Figure 5:**
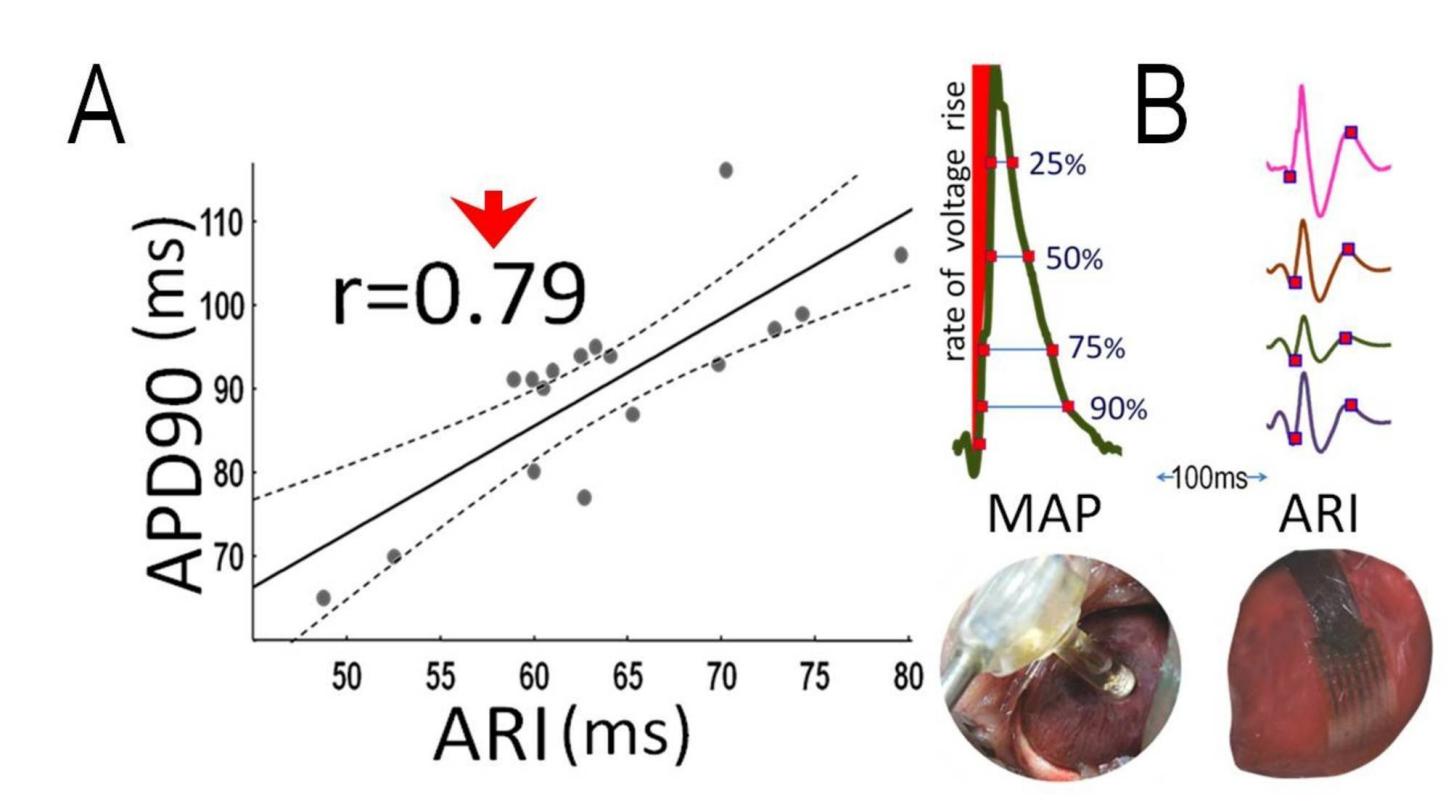
Action potential and activation recovery interval (A): Correlation between left ventricular action potential duration (APD) at 90% of repolarization (APD90) and activation recovery interval (ARI). (B) Examples of measurements

No variance was found between groups in either LV or RV APD. Likewise, no variance was present between groups in the ARI, which correlated with APD90 (r=0.79, p<0.001, figure 5A) and APD75 (r=0.54, p=0.018). Figure 5B illustrates examples of measurements from MAPs and ARIs.

**Figure 6:**
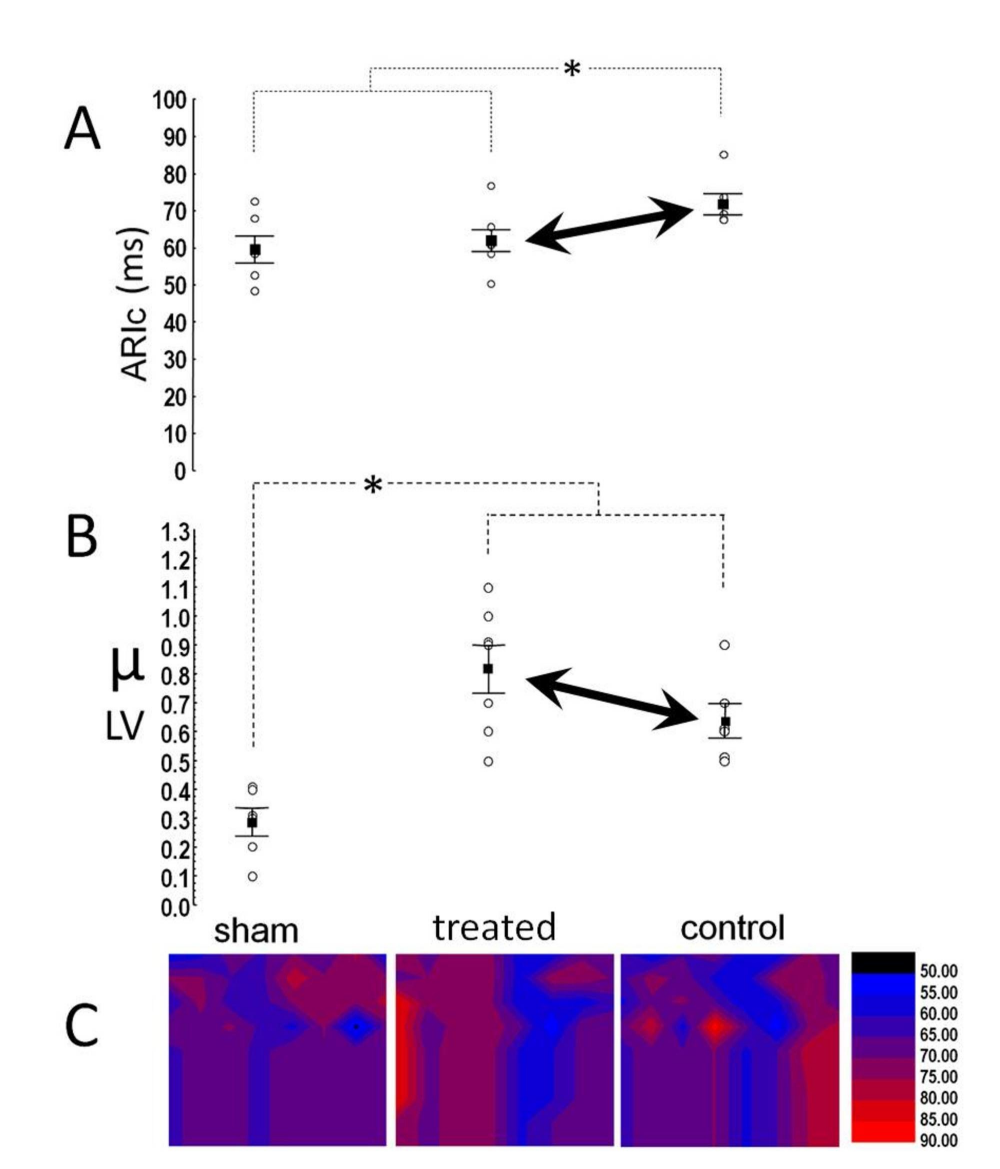
Repolarization duration and dispersion (A) Rate corrected activation recovery interval (ARIc) was shorter in treated rats but (B) shows repolarization dispersion tended to be higher. Asterisk denotes significant differences. (C): Examples of repolarization-maps

When corrected for HR, ARIc was shorter (p=0.046) in treated rats than in controls, and similar to that in sham-operated animals (Figure 6A). In the RV, no variance was present in either ARI or ARIc, and RV-ARI correlated with APD90 (r=0.63, p=0.008) and APD75 (r=0.47, p=0.047). Repolarization dispersion (μ) in the LV was higher in the MI groups than in sham-operated animals, but it was also marginally (p=0.082) higher in treated rats when compared to the controls (Figure 6B). Figure 6C displays representative examples of isochronal repolarization maps from the three groups; in the RV, no variance was present in μ.

## Discussion

Main findings

We characterized a cellularized scaffold that was subsequently implanted in the rats post MI. Cultures of hAT-derived MSCs revealed adequate differentiation capacity, fulfilling the criteria set by the International Society for Cellular Therapy [20]. We also observed a tubular structure formation, in keeping with the previously demonstrated plasticity of hAT-MSCs [21]. To enhance the angiogenic properties of the final construct, the scaffold was cellularized with a bi-culture, incorporating hAT-MSCs and HUVECs, according to previous suggestions [22]. Indeed, we observed early co-localization of HUVECs and hAT-MSCs in tubular sprouts, which formed a rapidly expanding vascular network. Additionally, another important feature of our scaffold, composed of low and high molecular weight alginate, is caused by its physical properties; specifically, our viscosity measurements demonstrated low values in liquid form, enabling smoother delivery by injection, coupled with optimal post-gelation viscosity.

The implantation of cellularized scaffolds by intra-myocardial injections was uneventful in all animals, consistent with previous experience [12]. We opted to implement the treatment shortly post MI, aiming at preventing, rather than reversing, LV remodeling [1]; should this approach surface into clinical trials, it can be combined with percutaneous coronary revascularization, thus maximizing the efficacy of both procedures. We examined the electrophysiologic effects of treatment two weeks post-implantation; at this time-point, fibrillar collagen accumulation is abundantly evident not only in the mature infarct scar, but also at remote regions of the rat myocardium, corresponding to human findings in the remodeled LV [23]. We report a comparable incidence of induced VTs in treated animals and controls; furthermore, no major differences were apparent in the electrophysiologic milieu, as shown by MAP recordings and activation mapping. Thus, our findings refute the hypothesis of potentially harmful synergistic effects on the ventricular electrophysiology of cellular and biomaterial implantation, when combined in a cellularized scaffold.

Inducibility of VTs

The arrhythmia score was similar between treated animals and controls, consistent with findings in rats transplanted with bone marrow-derived MSCs [7]; likewise, the induced VTs were comparable in the two groups, with rates similar to those reported after bone marrow mononuclear cell transplantation in rats [6]. By contrast, the arrhythmia score [7] and the rates of induced VTs [6] were higher after transplantation of skeletal myoblasts in these studies, strongly suggesting the dependence of the electrophysiologic alterations on the type of transplanted cells. This inference is further supported by findings in a porcine model, in which the incidence of inducible VTs after transplantation of AT-derived mononuclear cells did not differ from controls [16]. Moreover, the absence of arrhythmogenic effects in our experiments is also consistent with the low incidence of VTs in the clinical setting, after intracoronary administration of autologous bone marrow MSCs post MI [24]. Nonetheless, the transplantation of bone marrow-derived MSCs in rats resulted in less favorable electrophysiologic properties and higher inducibility of VTs, when compared to cardiac stem-cells, although a control arm was absent [25].

Local conduction

Slow conduction at the infarct border, a well-studied arrhythmogenic mechanism in healed MI [26], has been brought forward as an important parameter, responsible for the proarrhythmic effects of skeletal myoblast transplantation [3]. This view was corroborated by co-cultures of skeletal myoblasts and cardiomyocytes, demonstrating multiple areas with decreased conduction that formed the conditions for reentrant circuits [27]. These observations were further confirmed in-vivo in rats, in which localization of skeletal myoblasts in discrete clusters produced areas of delayed conduction at the infarct border [7]. Conduction abnormalities may also occur with MSCs, as noted in co-cultures of human MSCs with neonatal rat ventricular cardiomyocytes [4]; these abnormalities were attributed to a possible source-sink effect, with inexcitable MSCs acting as a current sink in a cardiomyocyte source, thus forming abnormal electrical loads that impede conduction. Nonetheless, this assumption was refuted in vivo, based on the absence of conduction defects after transplantation of bone marrow-derived MSCs in rats [7].

Theoretically, the risk of impaired propagation of electrical depolarization may be higher after the injection of biomaterials, which lack conductive properties, rather than after cellular treatments; however, the data on this field are scarce. Our findings do not validate this hypothesis, as the implanted scaffold, containing a low-viscosity hydrogel, did not affect electrical conduction at the border zone. This is consistent with recent results, showing normal conduction after the implantation of a polypyrrole polymer in rats one week post MI [17]. Two further studies in rats [14-15] focused on the acute electrophysiologic sequelae of scaffold implantation. More specifically, a poly (ethylene-glycol) hydrogel did not affect the propagation of electrical depolarization, except for cases of injection of high-viscosity material [15]; likewise, injections of low-viscosity alginate did not alter the incidence of VTs over a 24-hour observation period post-MI [14]. Thus, examined in the context of previous reports [14-15,17], our findings indicate the absence of major effects caused by biomaterials on electrical propagation and arrhythmogenesis, provided that their physical properties are kept optimal.

An important determinant of electrical propagation is membrane excitability which, in turn, depends on the availability of sodium channels at the onset of depolarization [28]. As part of the remodeling process, reduced sodium current has been demonstrated at the infarct border, secondary to decreased channel synthesis [29]. In keeping with these findings, the voltage rise at the MI border was lower in our MI groups, when compared to sham-operated animals; however, the values were similar in treated rats and controls, further supporting the absence of proarrhythmic effects of the implanted cellularized scaffold.

Repolarization duration and dispersion

Prolonged repolarization favors the development of afterdepolarizations that trigger VTs [30], signifying an important arrhythmogenic mechanism. Repolarization was shorter in our treated animals than in controls, consistent with the findings after bone marrow-derived MSCs were transplanted, and attributed to decreased Kir2.1 gene expression [9]. However, our cellularized scaffold did not prevent repolarization dispersion, which in fact, tended to be higher in treated rats. The explanation for this diverse response is unclear, and may partly account for the neutral effect of treatment on VT inducibility. Nevertheless, this finding may raise some safety concerns, and calls for further investigation; this statement is underscored by previous observations in the porcine model, in which a proarrhythmic substrate was demonstrated after intravenously infused MSCs post MI, although no data were provided on the inducibility of VTs [5].

Our results contradict to some extent those reported in rats two weeks after transplantation of bone marrow-derived MSCs, in the setting of acute MI [8]. In this study, a markedly prolonged APD was observed in MAP recordings at the infarct border in treated rats; however, perhaps paradoxically, this was associated with lower incidence of induced VTs [8]. Differences in the MSCs utilized may account for these discrepant results, but methodological issues in PES protocol appear as a more likely explanation.

Strengths and limitations of the study

We feel that our work adds to the evaluation of cardiac repair strategies post MI, focusing on the safety issues at a time point corresponding to the completion of LV remodeling. To enhance the translational value of our results, enalapril was given to all animals, as such treatment mitigates LV remodeling and has become part of standard post MI management [18]. Lastly, we comprehensively investigated several parameters characterizing the electrophysiologic milieu, aiming at identifying various underlying mechanisms. Despite these merits, two limitations should be acknowledged: first, the mortality data reported here should be viewed with caution, as the small number of our animal cohort does not provide sufficient validity to detect meaningful differences in mortality. Second, the main limitation of our work lies in our incomplete histologic evaluation of the extent of fibrosis and on gap junctional remodeling; hence, these data were not included in the present report.

## Conclusions

We characterized an alginate-based scaffold, seeded with hAT-MSCs and HUVECs. Two weeks post MI, a neutral effect was seen in the inducibility of VTs by PES. Likewise, conduction delays at the infarct border and voltage rise in MAP recordings were comparable between the two groups. No major differences were seen in repolarization duration, which was in fact, shorter in treated rats, when corrected for HR; however, a trend was noted toward enhanced repolarization dispersion. Our results indicate a low medium-term arrhythmogenic potential of the implanted cellularized scaffold. Further studies addressing safety issues are warranted, encompassing larger animal populations and longer observation periods.

## References

[1] Agathopoulos S, Kolettis TM Novel strategies for cardiac repair post-myocardial infarction. Curr Pharm Des.

[2] Menasche P (2003). Skeletal muscle satellite cell transplantation. Cardiovasc Res.

[3] Kolettis TM (2006). Arrhythmogenesis after cell transplantation post-myocardial infarction: four burning questions and some answers. Cardiovasc Res.

[4] Chang MG, Tung L, Sekar RB (2006). Proarrhythmic potential of mesenchymal stem cell transplantation revealed in an in vitro coculture model. Circulation.

[5] Price MJ, Chou CC, Frantzen M (2006). Intravenous mesenchymal stem cell therapy early after reperfused acute myocardial infarction improves left ventricular function and alters electrophysiologic properties. Int J Cardiol.

[6] Fernandes S, Amirault JC, Lande G (2006). Autologous myoblast transplantation after myocardial infarction increases the inducibility of ventricular arrhythmias. Cardiovasc Res.

[7] Mills WR, Mal N, Kiedrowski MJ (2007). Stem cell therapy enhances electrical viability in myocardial infarction. J Mol Cell Cardiol.

[8] Wang D, Zhang F, Shen W (2011). Mesenchymal stem cell injection ameliorates the inducibility of ventricular arrhythmias after myocardial infarction in rats. Int J Cardiol.

[9] Lai PF, Panama BK, Masse S (2013). Mesenchymal stem cell transplantation mitigates electrophysiological remodeling in a rat model of myocardial infarction. J Cardiovasc Electrophysiol.

[10] Kolettis TM, Vilaeti A, Dimos K, Tsitou N, Agathopoulos S (2011). Tissue engineering for post-myocardial infarction ventricular remodeling. Mini Rev Med Chem.

[11] Vilaeti AD, Dimos K, Lampri ES (2013). Short-term ventricular restraint attenuates post-infarction remodeling in rats. Int J Cardiol.

[12] Daskalopoulos EP, Vilaeti AD, Barka E (2015). Attenuation of post-infarction remodeling in rats by sustained myocardial growth hormone administration. Growth Factors.

[13] Yu J, Du KT, Fang Q (2010). The use of human mesenchymal stem cells encapsulated in RGD modified alginate microspheres in the repair of myocardial infarction in the rat. Biomaterials.

[14] Kontonika M, Barka E, Daskalopoulos EP (2014). Effects of myocardial alginate injections on ventricular arrhythmias after experimental ischemia-reperfusion. Trends Biomater Artif.

[15] Suarez SL, Rane AA, Munoz A (2015). Intramyocardial injection of hydrogel with high interstitial spread does not impact action potential propagation. Acta Biomater.

[16] Fotuhi P, Song YH, Alt E (2007). Electrophysiological consequence of adipose-derived stem cell transplantation in infarcted porcine myocardium. Europace.

[17] Mihic A, Cui Z, Wu J (2015). A conductive polymer hydrogel supports cell electrical signaling and improves cardiac function after implantation into myocardial infarct. Circulation.

[18] Nguyen T, El Salibi E, Rouleau JL (1998). Postinfarction survival and inducibility of ventricular arrhythmias in the spontaneously hypertensive rat: effects of ramipril and hydralazine. Circulation.

[19] Kolettis TM, La Rocca V, Psychalakis N (2016). Effects of central sympathetic activation on repolarization-dispersion during short-term myocardial ischemia in anesthetized rats. Life Sci.

[20] Dominici M, Le Blanc K, Mueller I (2006). Minimal criteria for defining multipotent mesenchymal stromal cells. The International Society for Cellular Therapy position statement. Cytotherapy.

[21] Planat-Benard V, Silvestre JS, Cousin B (2004). Plasticity of human adipose lineage cells toward endothelial cells: physiological and therapeutic perspectives. Circulation.

[22] Lesman A, Habib M, Caspi O (2010). Transplantation of a tissue-engineered human vascularized cardiac muscle. Tissue Eng Part A.

[23] Sun Y, Zhang JQ, Zhang J, Lamparter S (2000). Cardiac remodeling by fibrous tissue after infarction in rats. J Lab Clin Med.

[24] Wollert KC, Meyer GP, Lotz J (2004). Intracoronary autologous bone-marrow cell transfer after myocardial infarction: the BOOST randomised controlled clinical trial. Lancet.

[25] Zheng SX, Weng YL, Zhou CQ (2013). Comparison of cardiac stem cells and mesenchymal stem cells transplantation on the cardiac electrophysiology in rats with myocardial infarction. Stem Cell Rev.

[26] Kolettis TM (2013). Coronary artery disease and ventricular tachyarrhythmia: pathophysiology and treatment. Curr Opin Pharmacol.

[27] Abraham MR, Henrikson CA, Tung L (2005). Antiarrhythmic engineering of skeletal myoblasts for cardiac transplantation. Circ Res.

[28] Kleber AG, Rudy Y (2004). Basic mechanisms of cardiac impulse propagation and associated arrhythmias. Physiol Rev.

[29] Pu J, Boyden PA (1997). Alterations of Na+ currents in myocytes from epicardial border zone of the infarcted heart. A possible ionic mechanism for reduced excitability and postrepolarization refractoriness. Circ Res.

[30] Isidoro Tavares N, Philip-Couderc P, Papageorgiou I, Baertschi AJ, Lerch R, Montessuit C (2007). Expression and function of ATP-dependent potassium channels in late post-infarction remodeling. J Mol Cell Cardiol.

